# Multiscale alterations in bone matrix quality increased fragility in steroid induced osteoporosis

**DOI:** 10.1016/j.bone.2015.11.019

**Published:** 2016-03

**Authors:** A. Karunaratne, L. Xi, L. Bentley, D. Sykes, A. Boyde, C.T. Esapa, N.J. Terrill, S.D.M. Brown, R.D. Cox, R.V. Thakker, H.S. Gupta

**Affiliations:** a Queen Mary University of London, School of Engineering and Material Science, Mile End Road, London E1 4NS, UK; b MRC Mammalian Genetics Unit and Mary Lyon Centre, MRC Harwell, Harwell Science and Innovation Campus, OX11 0RD, UK; c Core Research Laboratories, The Natural History Museum, London SW7 5BD, UK; d Queen Mary University of London, Barts and the London School of Medicine and Dentistry, Institute of Dentistry, E1 2AD, UK; e Diamond Light Source Ltd., Beamline I22, Diamond House, Harwell Science and Innovation Campus, Chilton, Didcot, Oxfordshire, OX11 0DE, UK; f Academic Endocrine Unit, Nuffield Department of Clinical Medicine, Oxford Centre for Diabetes, Endocrinology and Metabolism (OCDEM), University of Oxford, Churchill Hospital, Headington, Oxford OX3 7JL, UK; g Department of Chemistry, University of Sheffield, Dainton Building, Brookhill, Sheffield S3 7HF, UK

**Keywords:** Glucocorticoid induced osteoporosis, In situ micro mechanical testing, Synchrotron small angle X-ray scattering, Nanoscale deformation mechanisms, Multiscale imaging

## Abstract

A serious adverse clinical effect of glucocorticoid steroid treatment is secondary osteoporosis, enhancing fracture risk in bone. This rapid increase in bone fracture risk is largely independent of bone loss (quantity), and must therefore arise from degradation of the quality of the bone matrix at the micro- and nanoscale. However, we lack an understanding of both the specific alterations in bone quality n steroid-induced osteoporosis as well as the mechanistic effects of these changes. Here we demonstrate alterations in the nanostructural parameters of the mineralized fibrillar collagen matrix, which affect bone quality, and develop a model linking these to increased fracture risk in glucocorticoid induced osteoporosis. Using a mouse model with an N-ethyl-N-nitrosourea (ENU)-induced corticotrophin releasing hormone promoter mutation (*Crh*^− 120/+^) that developed hypercorticosteronaemia and osteoporosis, we utilized *in situ* mechanical testing with small angle X-ray diffraction, synchrotron micro-computed tomography and quantitative backscattered electron imaging to link altered nano- and microscale deformation mechanisms in the bone matrix to abnormal macroscopic mechanics. We measure the deformation of the mineralized collagen fibrils, and the nano-mechanical parameters including effective fibril modulus and fibril to tissue strain ratio. A significant reduction (51%) of fibril modulus was found in *Crh*^− 120/+^ mice. We also find a much larger fibril strain/tissue strain ratio in *Crh*^− 120/+^ mice (~ 1.5) compared to the wild-type mice (~ 0.5), indicative of a lowered mechanical competence at the nanoscale. Synchrotron microCT show a disruption of intracortical architecture, possibly linked to osteocytic osteolysis. These findings provide a clear quantitative demonstration of how bone quality changes increase macroscopic fragility in secondary osteoporosis.

## Highlights

• In steroid induced osteoporosis, the mineralized collagen fibrils exhibit lower stiffness and a more random orientation distribution.
• Load induced reorientation at the fibrillar level is reduced in steroid induced osteoporosis compared to wild type.
• Reduced and more heterogeneous mineralization at the microscale was accompanied by significant increases in porosity.

## Graphical abstract

**Figure:**
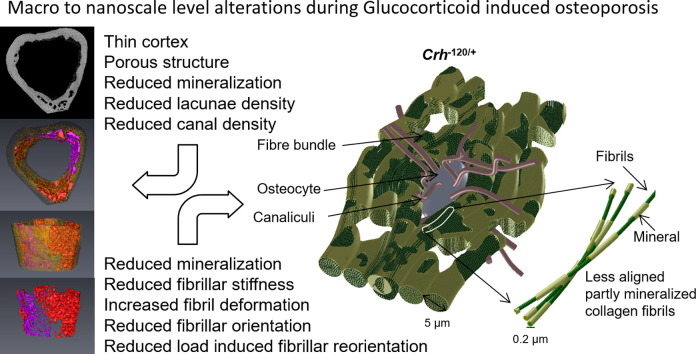

## 1 Introduction

Anti-inflammatory glucocorticoid treatments are mostly prescribed to an elderly population suffering from diverse disorders such as asthma, rheumatoid arthritis, immune diseases and following organ transplants [1]. However, glucocorticoid induced osteoporosis (GIOP), a form of secondary osteoporosis, is a clinically serious long term side effect of glucocorticoid treatment, resulting in loss in cancellous bone followed by cortical bone [2] and affecting 0.5% of the general population [3]. Osteoporotic fractures associated with glucocorticoid use occur in up to 30–50% of patients on chronic glucocorticoid therapy [4]. Further, GIOP is the most notable clinical skeletal disorder where the established paradigm of using only bone quantity to predict fractures is clearly insufficient to explain increased fracture risk, as bone mineral density (BMD) measurements show no significant association with fractures. GIOP patients have greater risk of fracture at higher BMDs when compared to postmenopausal osteoporotic women [1], [5]. It has been shown that glucocorticoid therapy affects both the amount of bone (bone quantity) as well as the micro-architecture and material level properties (local elastic modulus around osteocytes [6], crystal orientation and the degree of mineralization [6], [7] of the matrix) (bone quality) due to the down-regulation of bone-forming osteoblasts with concurrent alteration in the bone remodeling cycle [8]. Up to 40% reduction of both the mineral to matrix ratio and the elastic modulus (down to 14 GPa) was observed around osteocyte lacunae in [6], along with 18% reductions in trabecular bone volume, 12% lower trabecular connectivity and 7% lower trabecular number as measured with microcomputed tomography. However, the mechanisms by which these micro- and nanoscale changes in bone material quality lead to increased fracture risk in GIOP are currently unknown.

Deformation mechanisms at multiple structural levels between the nano- and the microscale – from the largest (osteons ~ 200 μm in diameter) down to the smallest (tropocollagen molecules and carbonated apatite crystallites) – lead to load-bearing bones achieving both a high stiffness and high work of fracture [9], [10], [11], [12]. Bone quality changes occur initially at the smaller length scales, dictated by rates of formation of new basic multicellular units by osteoblasts, at level of lamellae (~ 5 μm) and mineralized fibrils (~ 200 nm). Alterations in the bone extracellular matrix induced by glucocorticoid therapy are downstream effects of altered cellular activity of bone cells in the pathological condition and may be involved in the reductions of the global mechanical competence of bone. However, a gap exists both in our knowledge of the structural changes in GIOP [6], [7], and more significantly in the relation between such structural changes at the bone material level and the increased macroscopic fragility in GIOP. Therefore, there is a clear need to apply high-resolution imaging techniques to close the gap between onset of fracture relevant changes and diagnosis. We hypothesize that enhanced fracture risk in GIOP is associated with nanomechanical alterations at the fibrillar level, which link into larger scale deformation mechanisms. Here, to test this hypothesis, we combine multi-scale imaging techniques and mechanical testing on an animal model of GIOP.

For the animal model for GIOP, a recently published mouse model of endogenous hypercorticosteronaemia (Cushing's syndrome) was used [13], as the fracture risk associated with endogenous and exogenous GIOP have been shown to be similar [3]. The mouse model was generated via an N-ethyl-N-nitrosourea (ENU) induced mutation of the corticotrophin releasing hormone (*Crh*) promoter. The mutation, which involved a T-to-C transition at − 120 bp within the *Crh* promoter, resulted in increased transcription activity, and *in vivo* assessment of *Crh*^− 120/+^ mice revealed them to have obesity, hypercorticosteronaemia, hyperglycaemia, and low bone mineral density (BMD) when compared to wild-type (WT) mice. *Crh*^− 120/+^ mice, when compared to WT mice, showed reduction in mineralizing surface area, mineral apposition rate, bone formation rate and osteoblast number; this was also accompanied by an increase in adipocytes in the bone marrow. These phenotypic changes validate the used of the *Crh*^− 120/+^ mice as a model for Cushing's syndrome and GIOP [13].

In this study, the alterations in fibrillar-level deformability, mineralization and cortical micro-architecture in GIOP can thus be quantified and linked to macroscale mechanical properties using *in situ* X-ray nanomechanical imaging [14], [15] synchrotron micro-computed tomography and scanning electron microscopic investigations respectively.

## 2 Material and methods

### 2.1 Study design and cohort

*Crh*^− 120/+^ mice were identified in a dominant ENU mutagenesis screen at the MRC MGU Harwell [13]. Female *Crh*^− 120/+^ mice on a C57BL/6 genetic background (third generation) were used in all experiments; littermate WT mice were used as controls. Animals were 26 weeks of age at the time of sacrifice. Animals were anaesthetised before cervical dislocation; internal organs were removed from the body cavity and the whole body skeleton stored at − 20 °C until used.

### 2.2 Backscattered scanning electron (BSE) imaging

Quantitative BSE (qBSE) imaging was performed on transverse cross section at the mid-diaphysis to determine the microscale degree of mineralization. Mice femora (*Crh*^− 120/+^ = 5 and WT = 5) were sectioned into halves using a low speed diamond saw before dehydrating in ethanol and embedding in poly-methyl-methacrylate (PMMA) [16], [17]. Digital BSE imaging was performed with an Inspect-F, FEI scanning electron microscope equipped with an annular solid state BSE detector. The electron beam was adjusted to 20 kV accelerating voltage and 160 μA sample current was used to perform the analytical imaging. The working distance in the SEM was adjusted to 15 mm. The pixel resolution of the Digital BSE images from the midshaft transverse cross section was 0.3125 μm 1024 × 943 pixels) with gray level resolution of 256. These gray levels values were converted into calcium weight% values using carbon and aluminum (Micro-Analysis Consultants Ltd., Cambridgeshire UK) as gray level references. In *Crh*^− 120/+^ mice bone, two distinct regions (cavity region in the endosteal cortex and periosteal cortex) were observed. Three (WT) to six (*Crh*^− 120/+^: 3 from endosteal cortex and 3 from periosteal cortex) regions of interest (50 × 50 μm) from each BSE image (*Crh*^− 120/+^ = 5 and WT = 5) of the anterior transverse cortex were used to produce the grey level histograms. Using BMDD histograms, the mean calcium weight percentage Ca_mean_ and full width at half maximum (FWHM) [18] were calculated.

### 2.3 Synchrotron X-ray computed tomography

Synchrotron radiation microCT was performed at the imaging beam line I13-2 at Diamond Light Source (Harwell, UK) to visualize the vascular canal network. Three tibiae specimens from three WT mice and three tibiae from three *Crh*^− 120/+^ mice were oriented with their longitudinal axis parallel to the rotation axis during scanning. The effective voxel size was 1.6 μm^3^, providing a spatial resolution of 3.2 μm and field of view of 4.2 × 3.5 μm. Tomographic scans were obtained using photon energy of 18 keV and exposure time was 0.1 s. For each 3D data set, a total of 3600 projections were acquired over a range of 180°. The Diamond Light Source in-house algorithm was used to reconstruct tomographic data and 3D volumetric visualization of tibiae mid diaphysis was created by segmentation tools in Avizo 3D software (Burlington, MA, USA). Image volumes at the mid diaphysis of 1.35 × 1.34 × 1.00 mm^3^ were used for further morphometric analysis. Using Aviso intracortical cortical lacunae and canals were segmented from dense cortical tissue with simple thresholding due to their densities being significantly different, and this also provided volume measurements of the bone. STL meshes were created for segmented volumes (lacunae and canals) and imported into Blender software (Blender Foundation, Amsterdam, Netherlands) where it was separated by loose parts (unconnected mesh components) to give a total count of lacunae, canals and artefacts. The artifacts largely occurred outside the bone so could be selected, isolated and counted in Blender (using the same method as above). The mesh was then imported into Meshlab (http://meshlab.sourceforge.net/) where components less than 1% of the total mesh size were removed with the “remove isolated components (with respect to diameter) algorithm” leaving mainly the canals which could then be manually counted. Both the artefact and canals counts were then subtracted from the total count to give the lacunae number. We derived morphometric measures for female *Crh*^− 120/+^ and wild-type littermates, including lacunae number density (number of lacunae/cortical tissue volume) and canal number density (number of lacunae/cortical tissue volume).

### 2.4 Sample preparation for *in situ* tensile testing with SAXD

Mouse femora (*Crh*^− 120/+^ = 6 and WT = 4) from female *Crh*^− 120/+^ and wild-type littermates were dissected, skinned and muscle tissue removed. Then the bones were systematically prepared [17] for in-situ tensile testing (see Supplementary information for full detail). Bone strips only from anterior sections of the femora were used in this experiment such that long axis of specimens are parallel to the femur. The average length, width and thickness of the gauge regions were 5.0 mm, 1.0 mm and 0.2 mm, respectively.

### 2.5 In-situ tensile loading with small angle X-ray scattering

Samples were loaded at a constant velocity of 1 μm/s (strain rate = 0.02%/s) in a custom-made micromechanical testing machine [17] in the path of a microfocus synchrotron X-ray beam at beamline I22, Diamond Light Source (Harwell, UK). A schematic of the experimental setup is shown in Fig. S1 (Supplementary data). Samples were maintained in physiological saline in a fluid chamber and strained at 10^− 4^ s^− 1^, with SAXD spectra taken every 0.05% tissue strain up to failure (~ 0.5–1.0%). X-ray wavelength λ was 0.8857 Å and beam cross section 10 μm × 12 μm. Sample to detector distance was 1.034 m, measured with a calibration standard (type I collagen). During the experiment, exposure time for each SAXD spectra was kept to approximately 1 s, limiting the total X-ray radiation dosage to 29.4 kGy to minimise the influence of the X-ray radiation on the bone mechanical properties [19]. The collagen fibril strain ε_f_ was measured from change of the centre position *q*_0_ of the third order reflection peak, as described previously [14], [15], [20]. 2D SAXD patterns were reduced to one dimensional profiles by radial integration over a 20° sector oriented parallel to the tensile loading axis (Fig. S1). Subsequently, the third-order meridional fibrillar reflections were fitted to Gaussians with a linear background term obtain peak position *q*_0_. Axial fibrillar periodicity *D* = 6*π* / *q*_0_, and fibril strain equals the percentage increases in D relative to the unstressed state. Tissue strain was measured by non-contact video extensometry of displacement of horizontal optical markers (Fig. S1C inset i) on the bone mid diaphysis. We consider only the elastic regime of bone deformation in this paper, and hence only data collected from the linear region was used for further analysis. The elastic region for each sample was identified using the baseline of > 10% reduction the slope of the stress strain curve, shown in Fig. S2 in supplementary information.

### 2.6 Degree of fibrillar orientation

To determine the degree of orientation of the collagen fibrils with respect to the loading axis, the full width at half maximum of the meridional 3rd order reflection I_c_(χ) was estimated from unstrained samples. To eliminate mineral scattering background in SAXD, the total azimuthal intensity I(χ;*q*_0_) at *q* = *q*_0_ and the azimuthal distribution of mineral-scattering I_m;c_(χ;*q* = ± 5)were calculated. I(χ; *q*_0_) was calculated by radially averaging the SAXD intensity in a narrow band around *q*_0_. I_m;c_(χ) was similarly calculated by averaging I(χ) at wave-vectors lower *q*_l_ and higher *q*_*h*_ (Fig. S1D a and c) than *q*_0_ (Fig. S1D b). I_c_(χ) is the difference between I(χ;*q*_0_) and I_m;c_(χ;*q* = ± 5). The angular intensity of the 3rd order fibril reflection I_c_(χ) is plotted in Fig. S1F. The intensity was fitted to a Gaussian function I(χ) = I_0_ exp(−((χ − χ_0_) / Δχ_0_)^2^ / 2), where χ_0_ is the centre of the intensity distribution and Δχ_0_ proportional to the width (FWHM). The average rates of collagen fibrillar reorientation were determined for WT = 4 and *Crh*^− 120/+^ = 6 by calculating the slopes of FWHM vs. tissue strain curves.

### 2.7 Statistical analysis

To compare nanomechanical and synchrotron X-ray micro-computed tomography results between WT and *Crh*^− 120/+^ mice, Student t-tests were performed. One way ANOVA test with post-hoc Tukey HSD test was performed on mean calcium weight percentage Ca_mean_ and full width at half maximum (FWHM) data to assess statistical significance between periosteal and endosteal regions of *Crh*^-120/+^ mice and their WT littermates. Excel 2007 (Microsoft Office 2007) was used for the Student t-test, ANOVA and post-hoc Tukey HSD test.

## 3 Results

### 3.1 BSE Imaging and quantitative analysis

**Fig. 1:**
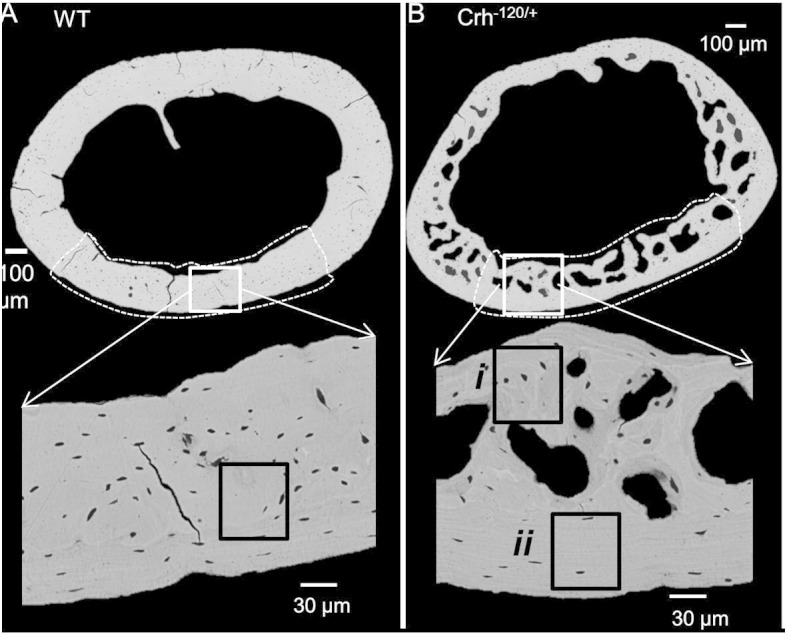
Backscattered scanning electron microscopy images collected from the mid shaft (transverse cross section) of the cortical bone of the WT (A) and *Crh*^− 120/+^ mice (B). Top images are full transverse cross section and areas surrounded by white dash lines represent cross section of mechanically tested sample. Higher magnification BSE images shown in the bottom panel are obtained from the anterior regions (white squares in top images) of the cortex. In *Crh*^− 120/+^ bone two distinct regions were observed. Dark gray sections (i) surrounded by white bands (cement lines) were observed near cavities in *Crh*^− 120/+^ bone and (ii) periosteal cortex (no cavities) were observed areas (50 × 50 μm) surrounded by black squares (i and ii) in bottom panel of A and B were used for quantification of mineralization parameters.

Backscattered scanning electron microscopy (BSE) was performed on femoral transverse cross sections to examine possible mineralization defects in *Crh*^− 120/+^ mice. Cortical microstructure of the *Crh*^− 120/+^ mice (Fig. 1B) femora was markedly different to the WT mice (Fig. 1A) cortical structure. BSE images of femoral transverse cross-sections of WT mice showed a uniform cortical thickness, whereas in the *Crh*^− 120/+^ mice, the posterior cortex was substantially thinner compared to the anterior cortex. The anterior, lateral and medial cross sections of *Crh*^− 120/+^ femora had a very large fraction of cavities. In contrast, WT femoral bone was uniformly dense around the full cortex. High magnification BSE images (Fig. 1A bottom) of the WT cortex show uniformly distributed lacunae. However, in *Crh*^− 120/+^ mice osteocytic area is low compared to WT mice. Strikingly, *Crh*^− 120/+^ mice cortices had numerous localized cement lines surrounding low mineralized tissue (Fig. 1B, lower image, (i)) near cavities, which were absent in WT cortices. These structures were ~ 50 × 50 μm in area, surrounded a significant number of osteocytes and localized to the endosteal cortex.

**Fig. 2:**
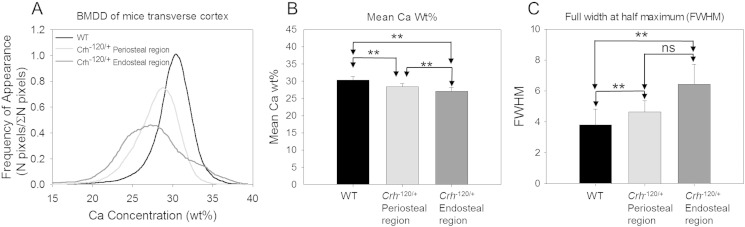
Quantitative backscattered scanning electron microscopy. (A) Bone mineral density distribution (BMDD) was produced for WT (black) *Crh*^− 120/+^ non-haloes (light gray) and *Crh*^− 120/+^ halo (dark gray). (B, C) Ca_mean_ (representing average mineral content) and FWHM (representing local variation of mineral content) calculated from BMDD and plotted for WT (black) and *Crh*^− 120/+^ non-haloes (gray) and halos (dark gray). Errors shown are standard deviations. One way ANOVA test results showed three groups were significantly different for Ca_mean_ and FWHM measurements. The post-hoc test (Tukey HSD test) was performed subsequently to identify which of the pairs of treatments are significantly different from each other. Pair-wise brackets denote statistical significance (**p < 0.01, ***p < 0.001, ns: not significant).

Bone mineralization density distribution (BMDDs) histograms (Fig. 2A) were plotted for *Crh*^− 120/+^ and WT femoral transverse cross-sections. Since we observed very distinct intra-tissue (regions surrounded by the localized cement lines) variation of mineralization on the *Crh*^− 120/+^ mice femora as reported above, both regions (Fig. 1Bi and ii) were used separately for quantitative BSE analysis. *Crh*^− 120/+^ mice showed a lowered average mineral content compared to WT (Fig. 1B). Mean calcium weight percentage (Ca_mean_) was lowest at regions (area surrounded by the cement lines) near the cavities in *Crh*^− 120/+^ mice (27.06 ± 1.18 S.D. (Fig. 2B), and significantly greater in the cortical periosteum (*p* < 0.01) at the regions away from cavities (28.44 ± 0.84 S.D.) (Fig. 1Bii). WT mice (30.32% ± 1.09 S.D.) had significantly higher (*p* < 0.01 for both WT vs. regions near and away from cavities in *Crh*^− 120/+^ mice) Ca_mean_ compared to *Crh*^− 120/+^ mice. The homogeneity of tissue-level mineralization (FWHM) was highest at regions around cavities (6.43 ± 1.30 S.D.) in *Crh*^− 120/+^ mice (Fig. 2C), and substantially lower in regions near the periosteal surfaces (4.63 ± 0.73 S.D.) away from the cavities. In contrast to *Crh*^− 120/+^ mice bones, WT mice had significantly lower (*p* < 0.01) FWHM (3.80 ± 1.02 S.D.).

### 3.2 Synchrotron X-ray micro-computed tomography

**Fig. 3:**
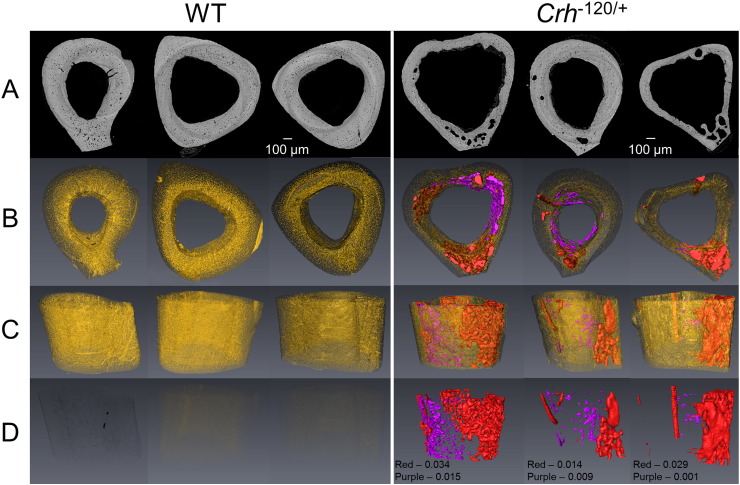
Synchrotron X-ray microCT transverse images (A) and 3D reconstructions (B, C and D) of WT (left) and *Crh*^− 120/+^ (right) tibiae mid diaphysis of three specimens. (A) Transverse SR CT sections (B) top view of 3D reconstructions of cavities (red), inner cortico-endosteal tissue (purple), canal and lacunae network (yellow) (C) Isometric projections of 3D reconstructions (transparency of canal and lacunae is set to 0.75) shows cavities and inner cortico-endosteal tissue in *Crh*^− 120/+^ specimens and higher number of canals and lacunae can be observed in WT tibiae than *Crh*^− 120/+^ bone (D) 3D reconstruction of cortico-endosteal pores and inner cortico-endosteal tissue can be only seen in *Crh*^− 120/+^. Volume fractions for cavities and inner cortico-endosteal tissue are given for *Crh*^− 120/+^ (transparency for canal and lacunae are set to 1).

**Fig. 4:**
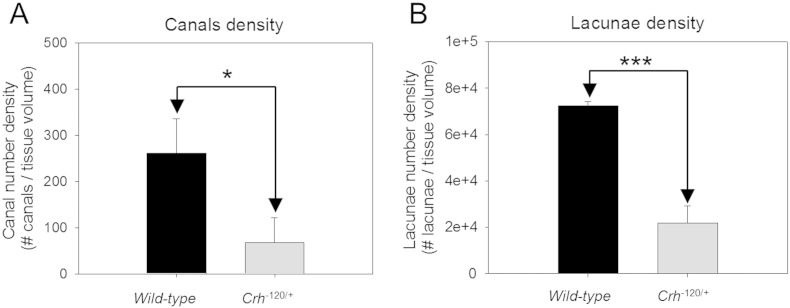
Morphometric analysis Synchrotron X-ray micro-computed tomography results (A) The canal network was characterized by the canal number density. (B) Measures for the lacunar system included the lacunar number density. Error bars are standard deviations. Student's t tests were used to compare canal density and lacunae density between wild-type and *Crh*^− 120/+^ mice. Pairwise brackets denote statistical significance (*p < 0.05, ***p < 0.001).

In order to gain better understanding of 3D distribution of cavities and micro structure of cortical bone, synchrotron X-ray micro tomography imaging was carried out. Results presented in Fig. 3C and D show most of these cavities (red; color online) are localized into the anterior cortico-endosteal bone along the tibiae shaft. These localized cavities are not present in WT mice. 3D reconstructions of vascular canal network and lacunae (segmented with yellow color; color online) presented in Fig. 3B show that WT and *Crh*^− 120/+^ bones have individual canals directly connected to the medullary cavity. However, WT bone exhibited very condensed network of canals and osteocyte lacunae homogenously distributed across the cortical bone. In contrast, in *Crh*^− 120/+^ mice most of the canal network and lacunae space have been replaced by cavities (Fig. 3B and C). Morphometric evaluation on vascular canals and osteocyte lacunae are shown in Fig. 4A and B respectively. These results indicated a significant reduction in canal density (*p* < 0.05) and lacunae density (*p* < 0.001) in *Crh*^− 120/+^ mice bones. Average volume fractions for porous sections (red segmented sections in Fig. 3) and residual regions (purple segmented sections in Fig. 3)) are 0.025 and 0.0083 respectively. In contrast, in WT mice no such structures were observed.

Furthermore, *Crh*^− 120/+^ mice bones exhibits some unmineralized tissue (Fig. 3A and segmented with purple color in Fig. 3B, C and D; color online) within the medullary cavity attached to the cortico-endosteal bone. This tissue has lower gray value (lower mineral content) compared to the cortical bone of *Crh*^− 120/+^ mice. The increased cavity structure (red voids; color online) in *Crh*^− 120/+^ mice bone is present across the entire length of the bone shaft, as evidenced from synchrotron X-ray microCT measurements across the entire mid-diaphysis (Supplementary Video S1, Video S2).

### 3.3 *In situ* tensile loading with synchrotron SAXD

**Fig. 5:**
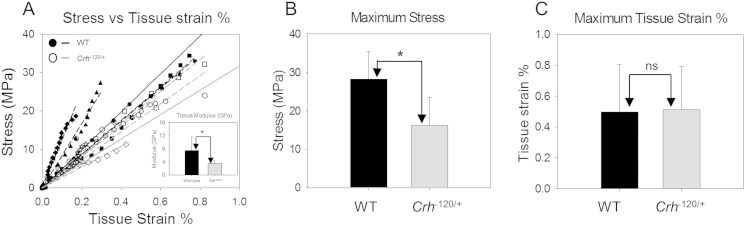
Macro mechanical testing results. (A) Stress vs. measured tissue strain % for WT (n = 4/filled symbols) and *Crh*^− 120/+^ (n = 6/open symbols) mice for each sample. Solid lines are average regression lines for wild-type (black) and *Crh*^− 120/+^ (grey). Average tissue modulus σ/ε_T_ showing in inset. (B) Maximum stress and (C) maximum tissue strain plotted for WT (black) and *Crh*^− 120/+^ (grey) mice. Error bars are standard deviations. Student's t tests were used to compare elastic moduli, maximum stress and maximum strain between wild-type and *Crh*^− 120/+^ mice. Pairwise brackets denote statistical significance (*p < 0.05, ns = not significant).

Tissue level and fibrillar and mechanics of cortical bone from femoral mid-diaphyses of 26 week old WT (n = 4) and *Crh*^− 120/+^ (n = 6) mice were measured using *in situ* micro mechanical tensile testing combined with microfocus SAXD. Porosity-corrected stress versus tissue strain was plotted (Fig. 5A) as a function of genotype. Tissue level elastic moduli are significantly (*p* < 0.05) lower in *Crh*^− 120/+^ mice compared to WT mice (Fig. 5A inset). Average yield stress (Fig. 5B) of the WT mice (28.2 MPa ± 7.1 S.D.) is significantly larger compared to *Crh*^− 120/+^ mice (16.2 MPa ± 7.3 S.D.). Tissue yield strain of *Crh*^− 120/+^ mice was not significantly different (*p* > 0.05) from WT mice (Fig. 5C).

**Fig. 6:**
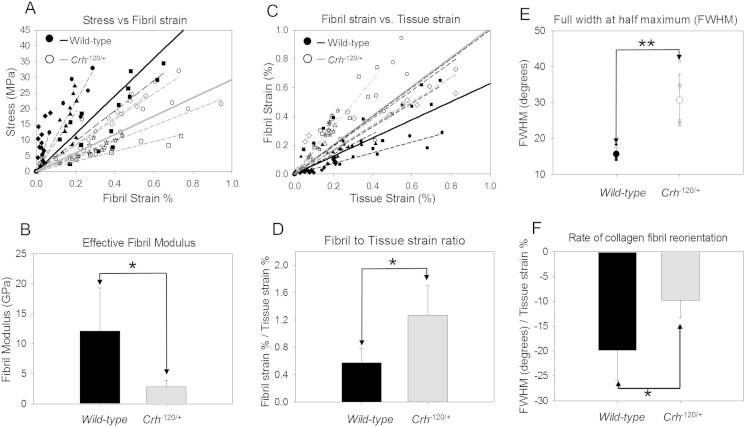
In-situ nano-mechanical and fibrillar orientation results. (A) Stress vs. fibril strain % for WT (n = 4/filled symbols) and *Crh*^*−* 120/+^ (n = 6/open symbols) (solid lines — r^2^ WT = 0.98 and r^2^*Crh*^*−* 120/+^ = 0.95). (B) Effective fibril modulus σ/ε_F_ plotted for elastic region of WT (black) and *Crh*^*−* 120/+^ mice (grey). (C) Fibril strain (ε_F_) % vs. tissue strain (ε_T_) % as a function of disease state WT (filled symbols) and *Crh*^*−* 120/+^ (open symbols) mice (solid lines — r^2^ WT = 0.88 and r^2^*Crh*^*−* 120/+^ = 0.87). (D) Average fibril strain to tissue strain ratio (ε_F_/ε_T_) plotted for elastic region of WT (black) and *Crh*^*−* 120/+^ mice (grey) (E) Change in fibril orientation (%) with loading vs. tissue strain (ε_T_) % as a function of disease state WT (filled symbols) and *Crh*^*−* 120/+^ (open symbols) mice (solid lines — r^2^ WT = 0. 96 and r^2^*Crh*^*−* 120/+^ = 0.82) Average rate of collagen fibrillar reorientation showing in inset. (F) Full width at half maximum of intensity distribution of the 3rd order fibril reflection was plotted for WT (filled symbols) and *Crh*^*−* 120/+^ (open symbols). Large symbols are average FWHM values and small symbols are individual data points. For A, C and E dash lines (WT = black and *Crh*^*−* 120/+^ = grey) are regression lines for each specimen. Solid lines are average regression lines for WT (black) and *Crh*^*−* 120/+^ (grey) of all test samples in each disease state. For B, D, E and F error bars are standard deviations. Student t-tests were used to compare fibril moduli, strain ratio, maximum fibril strain, rate of collagen fibrillar reorientation and FWHM between WT and *Crh*^*−* 120/+^ mice. Pair-wise brackets denote statistical significance (*p < 0.05, **p < 0.01, ***p < 0.001, ns: not significant).

Considering the fibrillar-level deformation, the gradient of stress versus fibril strain (Fig. 6A) in the elastic regime is denoted as the effective fibril modulus (E_f_ = dσ / dε_f_), as per our previous definition [17]. Average fibril modulus (Fig. 6B) shows a significant (*p* < 0.05) reduction of ~ 79% in *Crh*^*−* 120/+^ bone relative to WT. To calculate the fraction of tissue strain taken up at fibrillar level [15], fibril strain was plotted against macroscopic strain (Fig. 6C). Gradients of fibril strain (ε_F_) versus tissue strain (ε_T_) are clearly different (Fig. 6D), with the dε_F_/dε_T_ much higher in *Crh*^*−* 120/+^ mice compared to WT (1.18 ± 0.43) compared to 0.57 ± 0.2 S.D.) Maximal fibril strain for *Crh*^*−* 120/+^ specimens (0.63% ± 0.22 S.D.) was significantly higher (*p* < 0.05) compared to WT mice (0.3% ± 0.25 S.D.) (Data not shown).

The degree of fibrillar orientation (FWHM) between WT and *Crh*^*−* 120/+^ mice at unloaded state was significantly (*p* < 0.01) higher compared to WT, indicating a lesser degree of fibrillar alignment relative to the tensile axis (Fig. 6E). The load-induced fibrillar reorientation, the percentage change in fibrillar orientation (from the unloaded state for each sample) for WT and *Crh*^*−* 120/+^ mice shows the resultant change, indicating that the fibrillar orientation reduces for all samples (Fig. 6F), but is much less pronounced than *Crh*^*−* 120/+^ mice relative to WT mice. Rate of fibrillar reorientation with fibrillar deformation in *Crh*^*−* 120/+^ mice is significantly lower (*p* < 0.05) compared to WT mice.

## 4 Discussion

Here, we have applied a combination of nano- and microscale structural and mechanical probes to quantify the mechanisms by which bone material quality changes in a mouse model of GIOP [13] leads to increased macroscopic fragility. Glucocorticoid-induced osteoporosis [21], [22] is an especially appropriate osteoporosis model to clarify the mechanistic role of bone quality, as it is well-established (e.g. in postmenopausal women [23]) that the steroid-increased increase of fracture risk appears uncorrelated to changes in bone quantity. Nonetheless, while cellular changes in bone metabolism have been identified in GIOP [2], [6], less is known about the alterations in bone material [24], [25], and very little about the altered deformation mechanisms of the bone material in GIOP. We used X-ray nanomechanical imaging techniques combined with micro-structural probes of mineral content and 3D microarchitecture to provide a quantitative link between structure at the nano- and microscale and the mechanical quality deterioration in a mouse model for Cushing's syndrome, with relevance for GIOP.

While the mouse model used in this study exhibits endogenous steroid production characteristic of Cushing's syndrome [13], which is in contrast to the usual etiology of GIOP where steroids are administered exogenously as part of anti-inflammatory medication [1], there are sufficient similarities to make it a worthwhile comparison. The *Crh*^*−* 120/+^ mice in this study have been previously shown [13] to exhibit osteoporosis, specifically showing reduced bone formation, number of osteoblasts, mineral apposition rate, and fraction of the endosteal surface of cortical bone which was covered by osteoblasts. Further, an increased adipocyte concentration in *Crh*^*−* 120/+^ mice [18] suggest that bone marrow stromal cells preferentially differentiate to adipocytes rather than osteoblasts, as seen in GIOP [11], [26] In addition, atomic force microscopy and other imaging methods have shown [6], [24], [27] that GIOP cortical murine bone exhibits “haloes” of lesser mineralized tissue around osteocyte lacunae. Similar microstructural alterations are observed in the current mouse model as well as significantly altered 3D microstructural architecture, which gives confidence that the alterations in bone matrix mechanics and structure visible here are relevant to the case of GIOP. As discussed earlier [13], the current model can hence be considered a complement to existing models of exogenously induced GIOP [2], [28], [29] with special application to understanding the longer-term effects of GIOP on bone structure and quality, in line with the continuous production of steroids over the lifetime of the animal.

The main findings of our study can be summarized as follows:

• Nanoscale mechanical alterations: We observed a reduced fibril modulus, increased fibrillar extensibility, increased randomness of fibril texture and reduced rate of fibrillar reorientation in the cortical bone of *Crh*^*−* 120/+^ mice subjected to tensile loading, compared to WT controls
• Microscale material and structural alterations: A reduced average mineral content and increased heterogeneity of mineralization were accompanied by significant increases in porosity and alterations in 3D microarchitecture, presence of lower mineralized tissue around these pores and disrupted endosteal structure in the cortices of *Crh*^*−* 120/+^ mice compared to WT controls.
• Macromechanical changes: The tissue-level stiffness reduced, the maximum tissue strain increased and the breaking stress reduced also reduced in the bones of *Crh*^*−* 120/+^ mice compared to WT controls.

In the following, we will discuss these findings, and their relation to existing knowledge about the alteration of bone structure in GIOP as well as in related disorders like Cushing's syndrome in more detail.

At the microstructural level, the microCT images indicate substantial, interconnected pores inside the cortex of bones, which is accompanied by the reduction in mineralization of the remaining matrix. The open question is whether this difference is the product of disrupted endosteal structure, or due to the presence of blood vessels inside these cavities, leading to Haversian-type remodelling with secondary tissue formation. From prior histochemical studies of this model [13], it is found that due to a combination of reduced osteoblast coverage on the cortico-endosteal surface, a lower mineralizing rate and lower overall number of osteoblasts, the endosteal surface develops with a ruffled, porous surface characteristic of cancellous bone, which are the large voids visualized by our microCT data. Further, there is no direct evidence of blood vessels inside these cavities from microscopy or SEM images, suggesting that secondary remodelling may not be playing a role. Thus, the first possibility is more likely. However, it should be noted that osteocytes inside already formed cortical bone have been proposed to be capable of resorbing formed bone (osteocytic osteolysis) by leaching and proteolytic process of osteocytes [6], [30], [31], [32]. While this view is contested [33] it is possible that enhanced removal of endocortical bone tissue by osteocytic osteolysis activated in GIOP [6]may also be a causative factor behind the large voids and cavities found, and at present we cannot conclusively exclude either possibility. The microstructure of the tissue toward the endosteal surface exhibit regions of low overall mineralization (Fig. 2B), which bear some resemblance to the lowered mineralized haloes observed in GIOP bone in [6], and also show some highly mineralized thin lines at their peripheries. The low mineralized zones also have a more heterogeneous distribution, as characterized by the larger FWHM in BMDD results (Fig. 2C). The overall reduction in mineral content and the increased heterogeneity in *Crh*^*−* 120/+^ (Fig. 1) may be linked to the adverse effect of glucocorticoid treatment on cellular activities.

Synchrotron X-ray microtomography data demonstrated that cavities are interconnected to each other along the longitudinal direction of the tibiae in *Crh*^*−* 120/+^ mice. Furthermore this 3D representation (Fig. 3 qualitatively) and morphometric evaluation (Fig. 4) showed that the *Crh*^*−* 120/+^ bones has reduced proportion of normal vascular network and less density of osteocyte lacunae compared to WT cortical bone. However, a much larger fraction of intracortical cavities are found, unique to *Crh*^*−* 120/+^ mice, which may be the late-stage result of enhanced osteocytic osteolysis inside the cortical shell [6], [24], [31], [32]. These findings are less apparent when investigating 2D-only images such as BSE imaging. Quantitative analysis of 3D morphological analysis such as canals density and lacuna density was performed in this study. Despite the limitation of small (3) number of samples capable of being measured in the limited synchrotron time, qualitatively and quantitatively significant differences in microstructure was observed between *Crh*^*−* 120/+^ and WT mice.

In terms of bone matrix mechanics, we observed significantly reduced (~ 50%) elastic modulus (Fig. 5A) and strength (Fig. 5B) in the femora of GIOP-exhibiting *Crh*^*−* 120/+^ mice at the macroscopic scale, which remain significant after correcting for elevated microstructural porosity in *Crh*^*−* 120/+^ (WT 2% and *Crh*^*−* 120/+^ 30%; Fig. S2). However, the origin of the material-level changes causing this mechanical deterioration may lie at either or both the micro (lamellar) and the ultrastructural length scale in the structural hierarchy. We find evidence that reduced stiffness at the fibrillar level plays an important role in this mechanical deterioration: the effective fibril moduli (σ/ε_F_) in *Crh*^*−* 120/+^ mice are significantly (*p* < 0.05) less stiff (~ 75%) than controls (Fig. 6A and B). We speculate that the alterations could be due to the reduced stiffness in extrafibrillar environment in *Crh*^*−* 120/+^ tissue. In the linearly elastic region of deformation (Fig. 5A), the externally applied tensile strain can to be divided into a tensile stretching of the mineralized collagen fibril together with deformation at larger length scales, which may include shear in the extrafibrillar matrix between fibrils [34] or between lamellae. Our *in situ* SAXD results show (Fig. 6C) that because the fibril modulus is lower, the maximum deformation in the mineralized fibrils in *Crh*^*−* 120/+^ is significantly higher (up to ~ 0.63%) relative to WT (up to ~ 0.3%).

The fibril-strain/tissue-strain ratio in the WT mineralized collagen fibrils is ~½ (Fig. 6D), consistent with previous *in situ* SAXD on bovine fibrolamellar bone, but in contrast, the fibril-to-tissue strain ratio for *Crh*^*−* 120/+^ bone is ~ 1, within experimental error. While an increase in fibril-strain/tissue-strain ratio in well-oriented bovine fibrolamellar bone was earlier explained by us [15] as due to increased mineralization in the extrafibrillar compartment, this mechanism clearly cannot hold for the osteoporotic *Crh*^*−* 120/+^ mice, because they exhibit significant reduction in degree of mineralization (Fig. 2B). As the fibril orientation distribution (reflecting lamellar level architecture) is significantly more random in *Crh*^*−* 120/+^ than in WT (Fig. 6E), we conclude that microscale inhomogeneity in lamellar level fibril orientation in *Crh*^*−* 120/+^ may be playing a significant role in the altered fibril-strain/tissue-strain ratio as well as in the altered macroscale mechanics. The alterations of macroscopic mechanics with fibril orientation is consistent with a recent study showing that microfibril orientation dominates the local elastic properties of lamellar bone [35], The alteration in fibril-strain/tissue strain ratio is quite consistent with the highly porous, heterogeneous mineralized matrix observed in Fig. 1B. In such a system, the local tissue strain (distinct from the global tissue strain ε_T_) in the more randomly oriented fibrils in *Crh*^*−* 120/+^ mice will be different when compared with WT mice.

Our results show (Fig. 6F) that the mineralized fibrils in both groups undergo a reduction in degree of fibrillar orientation on loading, which corresponds physically to a stress-induced alignment of the fibrils toward the loading axis. However, the rate of the fibrillar reorientation is different between *Crh*^*−* 120/+^ and WT with a significantly lower rate of reorientation in *Crh*^*−* 120/+^ mice. Prior to discussing these differences, however, the magnitudes of the changes in the width of the fibril angular distribution (Fig. 6F) deserve comment. For tissue strains of ~ 1–2% (and equivalent fibril strains of ~ 0.5–1%) we observe much larger percentage changes in the width, of the order of 10–20%. At first glance this finding of relatively large reduction in the width is a very surprising result, as it is expected that the percentage change of the angular distribution will be comparable to the percentage change of the fibrillar elongation, and not much larger. It is important to note that the large angular change is not related to the disease-phenotype — both WT and *Crh*^*−* 120/+^ specimens have comparable order of magnitude effects, and the *Crh*^*−* 120/+^ reorientation is actually lower (Fig. 6F). In order to exclude artefacts from our data analysis, we took special care to fit the angular intensity profile to a Gaussian without a baseline as we found (data not shown) that the introduction of an artificial baseline significantly affected (reduced) the width of the peak of the remaining Gaussian, and as a result the percentage reductions were even larger. Further, we kept the meridional width of the 3rd order reflection (used for radial averaging) large enough such that all the intensity in the peak was averaged, not just the intensity along the meridional peak position. Lastly (data not shown), to exclude the possibility that this large change was a characteristic of our mouse bone cortical specimen preparation protocol, a comparable analysis of the percentage change in fibril width for standard tissue types like the bovine fibrolamellar bone and antler cortical tested by us previously [20] show similarly large reductions of the order of ~ 10% for strains < 1%. We can therefore say with confidence that this effect is a real one which is characteristic of cortical bone of various types in our samples.

**Fig. 7:**
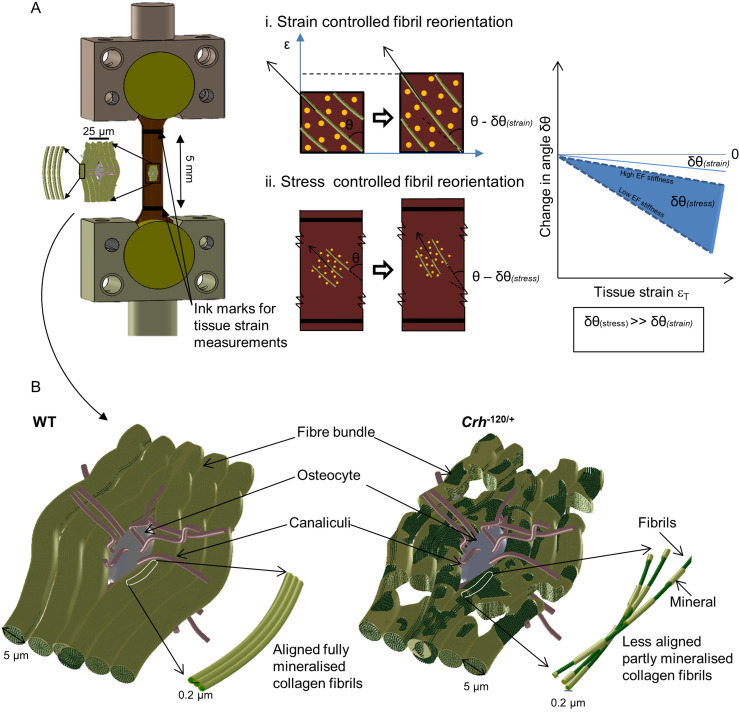
(A) Bone specimen mounted on tensile testing grips. Tissue (macro) level measurements were taken on a 5 mm long bone specimen. SAXD measurements were obtained from mineralized collagen fibrils at nano meter scale. (i) Strain controlled fibril reorientation–fibril reorientation for a measured localized tissue strain is δθ_(strain)_. (ii) Stress controlled fibril reorientation–fibril reorientation for a measured localized tissue strain is δθ_(stress)._ In this experiment fibril reorientation is occurred locally on the interrogated point on the sample, local stress-control instead of strain control (arrows on inset (i) and (ii) denotes fibril direction on tensile specimen. (B) Schematic representation of the bone matrix in WT and *Crh*^− 120/+^ conditions at the microscale and nanoscale. As our qBSE results only provide experimental information at the micrometre length scale, hence schematic was drawn with an osteocyte (10 to 15 μm) and several fibre bundles (5 to 10 μm). In WT mice, fiber bundles are fully mineralized, whereas *Crh*^− 120/+^ bone has micro pores and less mineralized. These less well mineralized structures are more localized around the osteocytes. At the fibrillar level we speculate that collagen fibrils are partly mineralized along the length of the fibril in the *Crh*^− 120/+^ condition. At this level, the WT mineralized collagen fibrils are fully covered with mineral. However, the mineralized fibrils from the *Crh*^− 120/+^ halo regions are partly mineralized (extra and/or intra) and less orientated. These structural alterations in *Crh*^− 120/+^ could be explained by the nano mechanical data obtained in this study.

In order to explain this large reduction, we need to consider the local loading environment of the fibril. If we assume (incorrectly, as it will turn out) that the fibrils and surrounding interfibrillar matrix are in an strain-controlled deformation mode, then it can be readily seen that the percentage change of angular position of the fibrils is of the same order as the fibril strain itself (Fig. 7A inset i), which is not what is observed. However, if we consider the fibrils to be relatively rigid fibers in a partially ductile interfibrillar matrix which transmits shear [20], it can be seen (Fig. 7A inset ii) that while the fibril strain can be small (due to the high axial stiffness of the rigid fiber) the reorientation of the fiber due to the resolved force perpendicular to the fiber can be significant (due to the lower stiffness of the extrafibrillar matrix). It is therefore clear that large percentage reduction in angular width of the fibril distribution, relative to the fibril strain, can be definitely possible in this case, and that the effect will be larger as the stiffness ratio between interfibrillar matrix and fibers increases. With this in mind, it is possible to consider the reduced rate of reorientation in GIOP as a possible alteration of the stiffness ratio between the mineralized fibrils and the extrafibrillar matrix, specifically to a stiffer extrafibrillar matrix and less stiff fibrils. In the absence of a detailed TEM level study of the type in [28], our discussion is speculative, but the reduced reorientation may indicate that the fibrils in GIOP are less completely mineralized (at the intrafibrillar level) than in WT, or that an excessive mineral deposition occurs outside of the fibrillar compartment.

We can also link the altered microstructure in *Crh*^*−* 120/+^ mice – specifically the demineralized structures (halos) around osteocytes (Fig. 1B) [24], the reduced frequency of osteocytes (Fig. 1, Fig. 3), and their qualitative shape – to the changed fibrillar mechanics and the altered loading environment in the bone tissue of *Crh*^*−* 120/+^ mice. Previous work has found that the geometrical properties and shape of osteocytes lacunae depend on age [36], anatomical site [37] and collagen fibre arrangement [38], with more elongated osteocytes in regions of greater collagen fibre alignment and rounder osteocytes in tissues with more random fibre orientation like woven bone. Here, our SAXD results showed that fibrillar orientation of *Crh*^*−* 120/+^ mice is lower (more random) than in WT mice (Fig. 6E), and also that the fibril modulus is lower (Fig. 6B). It is therefore likely that this alteration in the collagen fibril orientation and mechanics is linked to the change in osteocyte morphology to a less elongated structure [7], [24]. The alterations in intracortical porosity will most likely also play a significant role in reduction of mechanical competence, since extrinsic toughening mechanisms like crack bridging and crack deflection depend sensitively on the lamellar structure, orientation and mineralization [39], [40].These microstructural alterations may arise by three main mechanisms (i) crack path deflection at the interface between the lower mineralized halos and the surrounding tissue, or crack initiation at the cement lines around the halos, both of which will change the fracture toughness and (ii) a disrupted mechanosensory osteocytic network [27] and to the apoptosis of osteocytes [2], [41]. Fig. 7 shows some key elements in the alteration of the mineralized matrix in *Crh*^*−* 120/+^ mice, encapsulating the lower mineralization, more extensible and greater randomness of the fibrillar network.

## 5 Conclusion

In this study we demonstrated that in a mouse model for glucocorticoid-induced osteoporosis, both the fibrillar deformation mechanisms at the nanoscale and the microscale mineralization distribution are significantly altered compared to healthy bone. At the nanometre length scale, we found altered fibrillar deformation (increased extensibility and lower fibril modulus) in *Crh*^− 120/+^ mice bone, as well as less oriented fibrils. At the microscale in *Crh*^− 120/+^ mice, a lower mineral content, increased heterogeneity in mineralization near osteocytes and significant alterations in the three-dimensional mineralized matrix is observed. In contrast, WT bone is more uniformly mineralized as shown by the qBSE results. We propose that the altered deformation mechanisms at the nanoscale – increased flexibility, lower fibril modulus, altered fibrillar reorientation – in conjunction with altered microstructural toughening mechanisms due to heterogeneous mineralization are critical factors leading to the increased macroscopic fragility in GIOP.

The following are the supplementary data related to this article.

Supplementary material

Video S1 3D reconstruction of WT tibia mid diaphysis showing vascular network and distribution of osteocyte lacunae.

Video S2 3D reconstruction of *Crh*^− 120/+^ tibia mid diaphysis showing reduced vascular network and disturbed distribution of osteocyte lacunae. Resorption cavities can be observed along the entire length of the bone and they are segmented with red color for better visualization.

## Funding sources

Medical Research Council UK; Diamond Light Source Ltd., Diamond House, Oxfordshire, UK; School of Engineering and Material Sciences, Queen Mary University of London, London, E1 4NS, UK; Engineering and Physical Research Council (EPSRC) UK, Swindon, UK.

## Disclosure

The authors state that they have no conflicts of interest.

## Conflict of interest

All authors have no conflict of interest.
