# Supplementary material for: Multiscale alterations in bone matrix quality increased fragility in steroid induced osteoporosis
Source: Bone. 2016 Mar;84:15–24. doi: 10.1016/j.bone.2015.11.019 (PMC4764652; doi:10.1016/j.bone.2015.11.019)
Supplement: Supplementary file 1 — Supplementary material [file mmc1.doc]

**Supplementary information**

**Material and methods**

**Ethics statement**

All animal studies were carried out using guidelines issued by the Medical Research Council in 'Responsibility in the Use of Animals for Medical Research' (July 1993) and Home Office Project License Number 30/2642 Experiments were approved by the Medical Research Council Harwell ethics committee.

**Animals**

*Crh*^-120/+^ mice were identified in a dominant ENU mutagenesis screen at the MRC MGU Harwell . *Crh*^-120/+^ mice on a C57BL/6 genetic background (third generation) were used in all experiments; littermate wild type mice were used as controls. All animals were housed and maintained in the Mary Lyon Centre at the MRC Harwell, under specific pathogen-free conditions in individually ventilated cages, with environmental conditions as outlined in the Home Office Code of Practice. Animals were 26 weeks of age at the time of sacrifice. Animals were anaesthetised with sodium pentobarbitone (Pentoject, Animalcare, York, UK) before cervical dislocation; internal organs were removed from the body cavity and the whole body skeleton stored at -20°C until used.

**Sample preparation for in-situ tensile testing with SAXS**

Mouse femora (*Crh*^-120/+^ = 6 and wild-type = 4) from female *Crh*^-120/+^ and wild-type littermates were dissected, skinned and muscle tissue removed. Then the bones were longitudinally sectioned (**Figure S1A**) using a diamond saw under constant irrigation. Only anterior sections (**Figure S1C inset ii**) of the femora were used in this experiment. The ends of the bone strips were secured in water resistant dental cement (Filtek^TM^ Supreme XT, 3M ESPE, USA.) as described in our previous study to grip the samples in the tensile testing device. A previously developed custom made milling machine was used to machine the femurs in a stepwise fashion to form dumbbell shaped test specimens. Samples were clamped in a fluid chamber mounted on motorized linear stages (M110.1DG linear stages; Physik Instrumente, UK) and milled from the medial and lateral sides of the bone (**Figure S1B**) leaving reduced gauge width at the mid diaphysis by using a 0.8 mm diameter cutting tool (Dremel Engraving cutter product No. 111; Dremel Inc, Uxbridge, UK) connected to a fixed high-speed rotating milling tool (Dremel 300 series; Dremel Inc., Uxbridge, UK). A light microscope (SPEC) was used to capture the images of the milled samples and Image J software (Image J, NIH, USA) was used to measure the width and the thickness of each sample. The average length, width and thickness of the gauge regions were 5.0 mm, 1.0 mm and 0.2 (as shown by BSE images in **Figure S1C inset ii**) mm, respectively.

**Tissue strain measurements**

The samples were loaded at a constant velocity of 1 µm/second (strain rate = 0.02%/s) in a custom-made micromechanical testing machine up to failure (**Figure S1C**). Tissue strain was measured by non-contact video extensometry by imaging the separation of two horizontal lines that were marked (**Figure S1C** inset i) on the bone mid diaphysis surface. The tensile tester fluid chamber interior was illuminated by fixing a waterproof LED light within the chamber, which increased the contrast quality between marks and bone. Images were captured by a CCD camera (Basler Vision Technologies, Ahrensreide, Germany) viewing the chamber from the side (normal to the X-ray beam). These high resolution (1024 × 768) images were later analysed using an in-house LabVIEW/NI Vision-based digital image correlation package for tracking optical marker displacement to measure microstrains . While all samples were deformed to fracture, only the data from the linear (elastic) regime of deformation was used for analysis. The linear region was identified by noting where the transition to the inelastic regime took place from changes in tangent modulus. For each tissue strain value, the tangent modulus was calculated over a region of 0.1 % starting from that tissue strain value. The transition to the inelastic regime was defined as the point where the tangent modulus reduces 10% or less compared to its initial value (**Figure S2**).

**Porosity correction**

In order to correct the nominal stress values for the reduction in effective cross-sectional area (**Figure S1C inset ii**) due to microstructural porosity. The porosity fractions (WT – 3.5% ± 0.05 S.D. and *Crh*^-120/+^ - 29% ± 0.04 S.D.) for selected regions of interest (100 × 100 pixels each) was measured using BSE images (spatial resolution 2–3 µm) of the anterior cortex was determined using a standard plug-in BoneJ for the image processing program ImageJ (NIH, Bethesda, USA). Typical curves for nominal stress/tissue strain for one WT and *Crh*^-120/+^ mice are shown in **Figure S2**. Porosity-corrected stress values are hereinafter referred to as simply stress.

**Supplementary Figures**

**
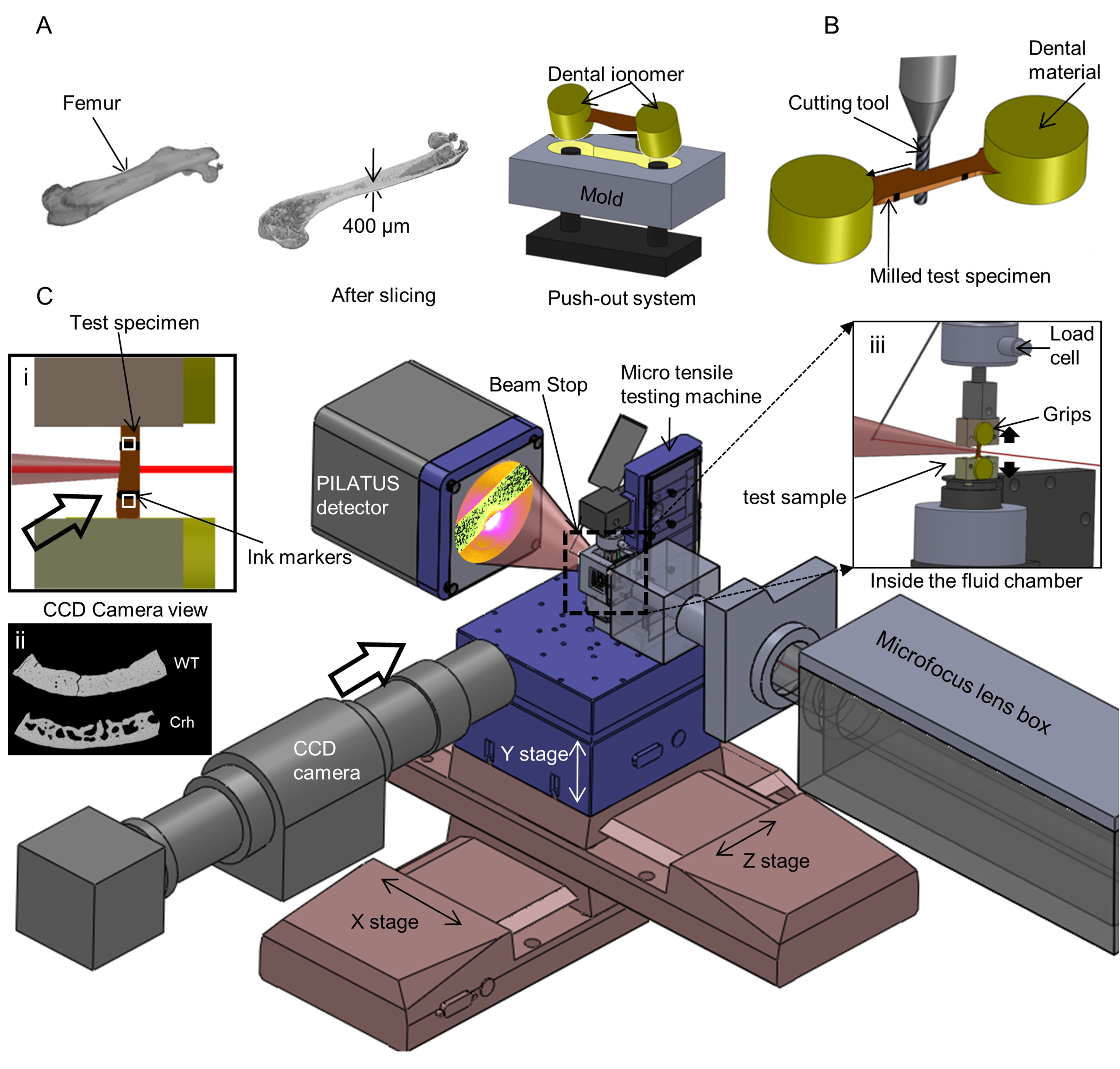
**

**Figure S1:** *Samples were systematically prepared for the in-situ mechanical testing combined with microfocus SAXD.* (A) Sample preparation procedure: Mouse femur was longitudinally sectioned using a low speed diamond saw. Using a mold and a push-out system Bony ends were secured in the dental ionomer . (B) Lateral and medial sides of the embedded bone sample were milled using 0.8 mm cutting tool (moving direction of the cutting tool was shown by the arrow). Black lines on the bone are reference markers for tissue strain measurements. (C) The experimental setup for the in-situ microtensile testing with microfocus SAXD at the I22 beam line, Diamond Light Source Ltd. The CCD camera views the sample at a 90° angle to the X-ray beam as not to block the beam path (inset i). BSE images of cross sections of tensile test specimens (WT and *Crh*^-120/+^) shown in the inset ii. Sample is immersed in the fluid chamber by securing in the grips as shown in the inset iii.


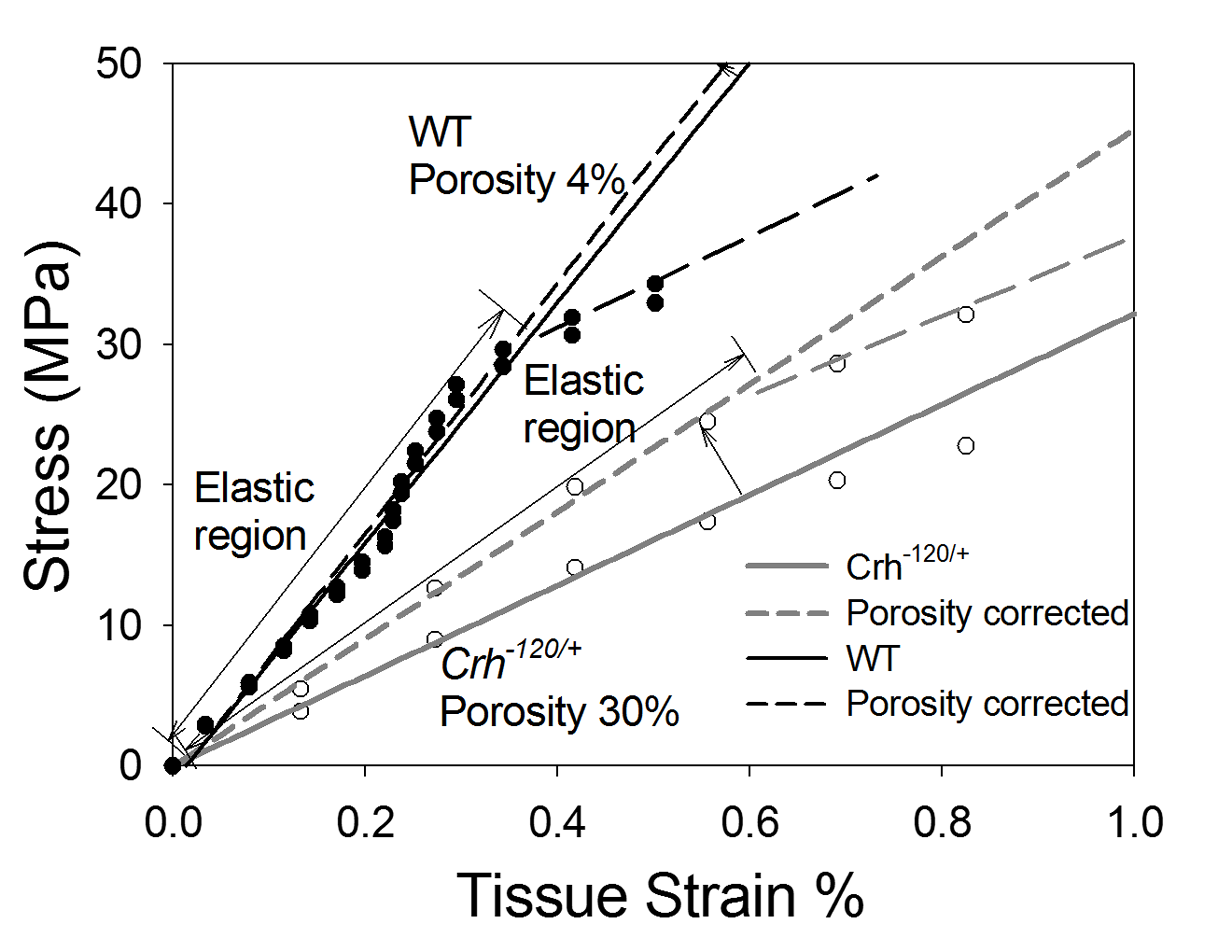


**Figure S2: *Porosity correction and criterion for linear region*** - Representative stress-tissue strain % curves are shown for the tensile tests of WT (filled symbols) and *Crh*^-120/+^ mice (open symbols). Solid (data not corrected for porosity) black and grey lines are linear regressions for WT and *Crh*^-120/+^ respectively. Porosity was estimated from BSE images of WT (4 %) and *Crh*^-120/+^ (30 %) transverse cross sections of mechanically tested bone samples. Stress vales were corrected for porosity and re-plotted on the same graph, corrected data indicated with dash lines. Yield point of each stress strain curve was determined by the data point where the elastic modulus (slope of the stress strain curve) changes by 10% (long dashed lines are guides to the eye)

**Figure S3**: (A) 2D SAXD pattern from a test specimen, showing the 3^rd^ order and 5^th^ order discrete reflection arising from the collagen D period, and the diffuse scattering intensity from the mineral nanocrystallites (B) Integrated intensity profile in the radial directions in the angular region (white solid lines) shown in A, showing the 3^rd^ and 5^th^ orders of the collagen reflections. (C) Same 2D SAXD pattern with the angular intensity distribution (bands were surrounded by black dash lines) of the 3^rd^ order fibril reflection I_c_(χ). This was measured by radially integrating the intensity in three narrow bands around *q*_0_. I_m, Inner_ (χ), I_m, Outer_ (χ) and I_c Middle_ (χ). The azimuthal intensity distribution of mineral-scattering I_m;c_(χ) were calculated by averaging I_m,Inner_(χ) and I_m, Outer_(χ). Intensity from the mineral scattering background was subtracted from intensity from the collagen D-period reflection (I_c Middle_ (χ)) (D) Mineral scattering background subtracted 3^rd^ order intensity profile and Gaussian fit curve

**Video S1**: 3D reconstruction of WT tibia mid diaphysis showing vascular network and distribution of osteocyte lacunae

**Video S2**: 3D reconstruction of *Crh*^-120/+^ tibia mid diaphysis showing reduced vascular network and disturbed distribution of osteocyte lacunae. Resorption cavities can be observed along the entire length of the bone and they are segmented with red to better visualisation.

1. Bentley L*, et al.* (2014) An N-ethyl-N-nitrosourea induced corticotropin-releasing hormone promoter mutation provides a mouse model for endogenous glucocorticoid excess. *Endocrinology* 155(3):908-922.

2. Karunaratne A*, et al.* (2011) Significant deterioration in nanomechanical quality occurs through incomplete extrafibrillar mineralization in rachitic bone: evidence from in-situ synchrotron X-ray scattering and backscattered electron imaging. *Journal of Bone and Mineral Research*:n/a-n/a.

3. Benecke G, Kerschnitzki M, Fratzl P, & Gupta HS (2009) Digital image correlation shows localized deformation bands in inelastic loading of fibrolamellar bone. *J Mater Res* 24:10.

4. Doube M*, et al.* (2010) BoneJ: Free and extensible bone image analysis in ImageJ. *Bone* 47(6):1076-1079.
