# Supplementary figures and images for: Multiscale alterations in bone matrix quality increased fragility in steroid induced osteoporosis
Source: Bone. 2016 Mar;84:15–24. doi: 10.1016/j.bone.2015.11.019 (PMC4764652; doi:10.1016/j.bone.2015.11.019)

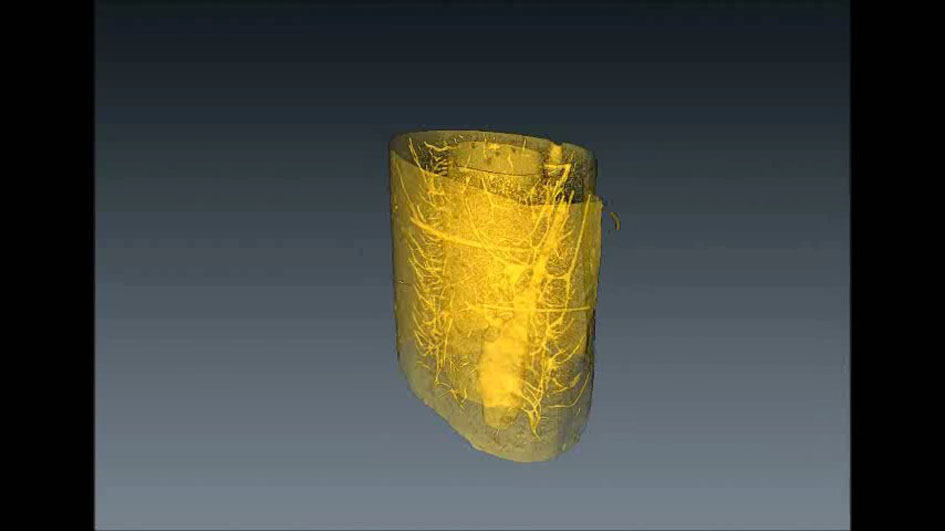

Supplement: Video S1 — 3D reconstruction of WT tibia mid diaphysis showing vascular network and distribution of osteocyte lacunae. [file mmc2.jpg]

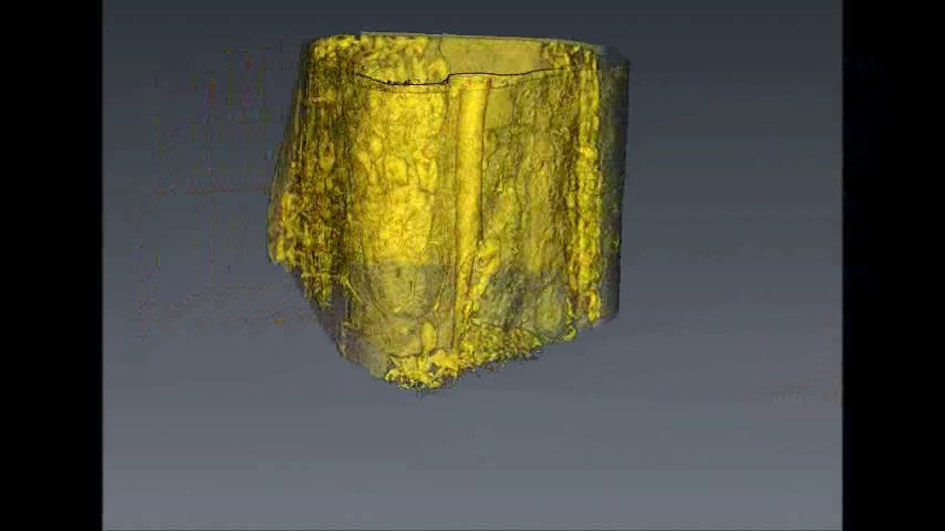

Supplement: Video S2 — 3D reconstruction of Crh− 120/+ tibia mid diaphysis showing reduced vascular network and disturbed distribution of osteocyte lacunae. Resorption cavities can be observed along the entire length of the bone and they are segmented with red color for better visualization. [file mmc3.jpg]
